# Comparison of Reflective Properties of Materials Exposed to Ultraviolet-C Radiation

**DOI:** 10.6028/jres.126.017

**Published:** 2021-08-20

**Authors:** Pawel de Sternberg Stojalowski, Jonathan Fairfoull

**Affiliations:** 1 Aseptium Limited, Solasta House, Inverness IV2 5NA, Scotland

**Keywords:** disinfection, light-emitting diodes, photochromic, polytetrafluoroethylene, reflectivity, scattering, ultraviolet-C

## Abstract

The reflectivity of material lining the inside of a disinfection chamber can have a dramatic effect on the ultraviolet-C (UV-C) radiation dose received across all sides of a contaminated object. Because minimum UV-C dosages are required to reliably inactivate microorganisms, it is crucial for the disinfection chamber to have either multiple UV-C sources or a highly reflective internal surface. This article describes an experimental comparison of four different materials, polytetrafluoroethylene (PTFE), acrylonitrile butadiene styrene, silver gloss self-adhesive aluminum, and Rosco matte black Cinefoil, to determine their efficacy as UV-C reflectors by using a custom-designed testing apparatus utilizing a UV-C radiation-emitting diode alongside photochromic UV-C indicators, allowing for a full 360° analysis of a target object and its received UV-C dose. Results determined that UV-C radiation received at the photochromic indicators varied greatly among the chosen materials, with PTFE providing the most uniform levels of radiation across all sides of the test object.

## 1 Introduction

Ultraviolet-C (UV-C) light is used in a variety of environments for the disinfection of surfaces and fluids. In order to reliably eliminate microorganisms, all sides of an object or all the fluid must be exposed to a sufficient inactivation dose. Because of the nature of light, its sources, and the three-dimensional (3D) character of the processed object, multiple light sources would have to be used to expose all sides evenly. Alternatively, a reflective material on the inside of a disinfection chamber can be used to reflect the light such that all surfaces of the object are exposed to its power evenly and sufficient disinfection dose is delivered to all surfaces of the processed object.

The reflective properties of the internal walls of a disinfection chamber are therefore crucial for effective UV-C radiation distribution. Surface reflections are described as either diffuse or specular, where diffuse reflection redirects incoming radiation randomly and uniformly in all directions, whereas specular reflection redirects incoming radiation in a single direction as determined by Fresnel's law [1]. For an opaque surface, energy that is not reflected is absorbed by the material [2, 3].

In most cases, microbial inactivation is a function of the total UV-C energy absorbed (given in millijoules), fluence rate (milliwatts per square centimeter), and the specific rate constant unique to each type of microbe (millijoules per square centimeter), where fluence rate is the "radiant power passing from all directions through an infinitesimally small sphere of cross-sectional area d*A*, divided by d*A*" [4].

Microbial inactivation is commonly described using the log-linear model [5], giving the fraction of living microbes after a treatment in relation to the inactivation rate constant for the microbe of interest and the delivered UV-C fluence.

This article describes an experimental study that analyzed the reflective properties of different materials exposed to UV-C radiation. It is intended to demonstrate a simple and practical method for comparison between different materials and guide those who develop UV-C disinfecting equipment. There is a scarcity of peer-reviewed literature on this subject, and our work is intended to help fill this gap to support the development of test methods and standards for UV-C disinfection.

## 2 Design of the Experiment

**Fig. 1:**
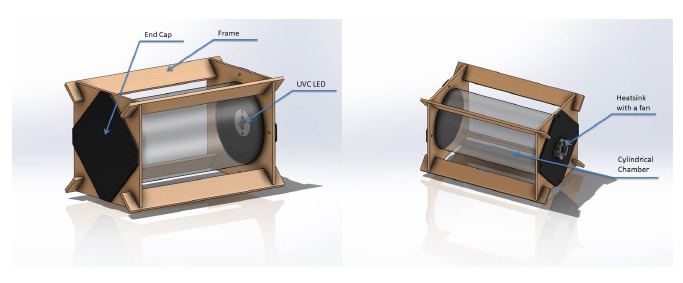
Experimental test chamber construction. LED = light-emitting diode.

In order to analyze how an object is exposed to UV-C radiation inside of a disinfection chamber (Fig. 1), a test apparatus was constructed to compare how the choice of different lining materials impacts the uniformity of the disinfecting dose on the sample disinfected object. To simulate a 3D disinfected object, a process challenge device was designed to be placed inside of the test chamber.

The idea behind the test apparatus was to create a worst-case scenario for uniformity of exposure, and so a single light source was used, exacerbating the need for the materials that cover the internal surfaces of the chamber to reflect and scatter the light effectively.

### 2.1 Test Apparatus

**Fig. 2:**
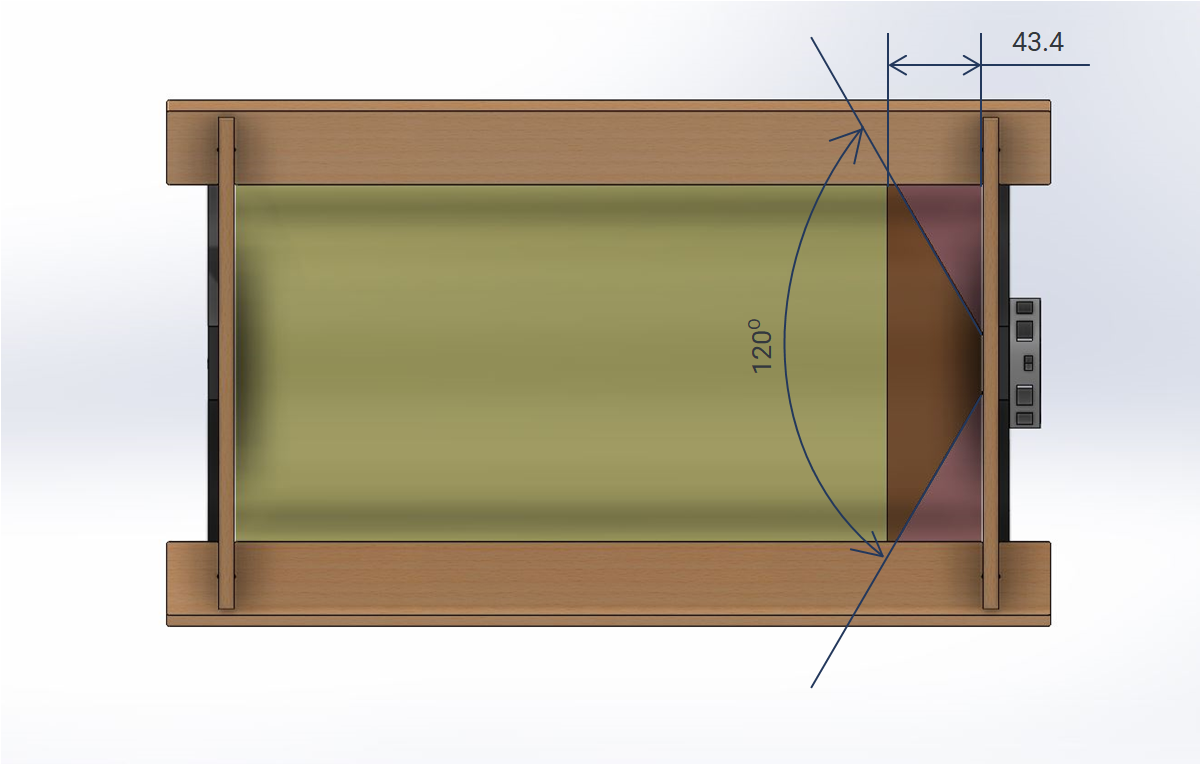
Direct (yellow) and indirect (purple) light exposure zones in the chamber.

For the purpose of the experiment, a cylindrical test chamber was constructed measuring 300 mm in length and 150 mm in diameter. The frame of the test chamber was designed such that different lining materials could be rolled up and formed into a cylinder that would fit into the frame as per Figs. 1 and 2. The ends of the cylinder were 3D printed from black acrylonitrile styrene acrylate (ASA) filament (Prusament ASA jet black [6]).^1^1 Certain commercial equipment, instruments, or materials are identified in this paper to specify the experimental study adequately. Such identification does not imply recommendation or endorsement by the National Institute of Standards and Technology, nor does it imply that the materials or equipment identified are necessarily the best available for the purpose. Both fit into the medium-density-fiberboard (MDF) frame such that a rolled sheet forming the wall of the cylinder slotted between the end cap and the frame, ensuring no light could escape the cylinder.

At one end of the cylinder, a single UV-C light-emitting diode (LED) (LiteON, LTPL-G35UVC275GH [7]) was installed centrally, emitting light to the inside of the cylinder. This LED was selected for its relatively high optical power output and wide viewing angle of 120°.

Because of the high power output, an aluminum heat sink with a fan was installed on the back of the LED's printed circuit board. This ensured that the diode could operate for prolonged periods of time (over 1 h intervals) at a stable temperature.

The 120° viewing angle of the LED ensured that upon installation, light was directed to the inside of the cylinder, such that direct exposure on the internal walls was achieved 43.4 mm from the end cap where the LED was situated. The internal wall of the end cap on which the LED was mounted would be exposed to only indirect light reflected from the other walls of the cylinder, as per Fig. 2.

### 2.2 Process Challenge Device

**Fig. 3:**
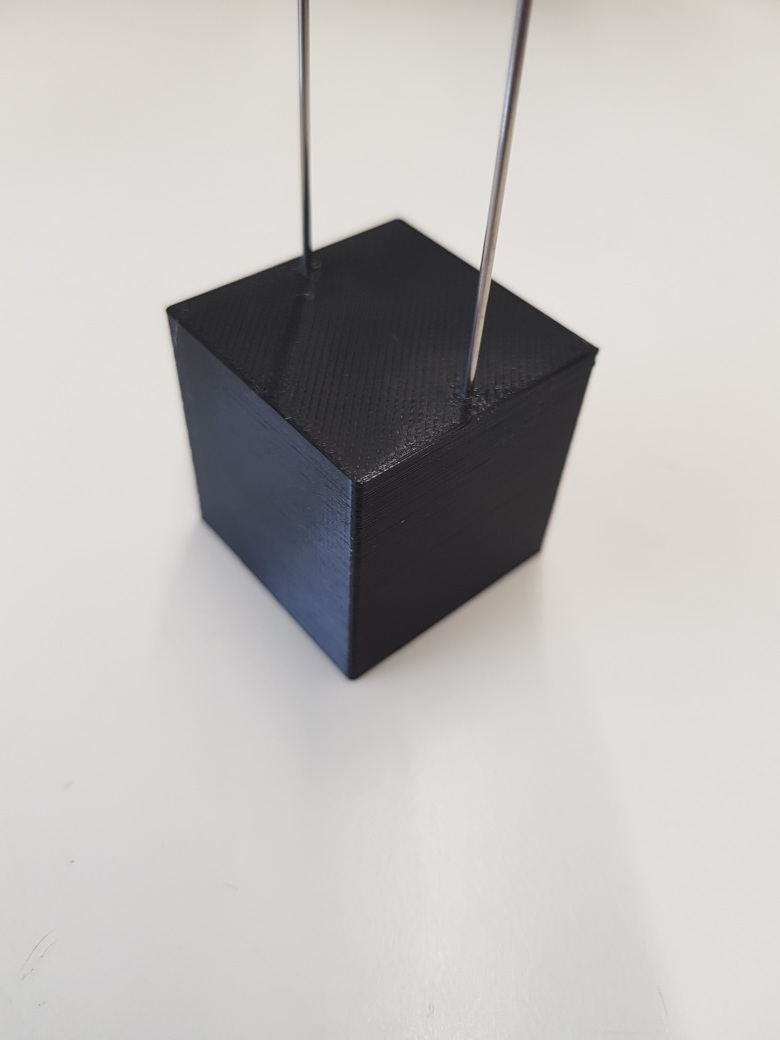
Process challenge device.

**Fig. 4:**
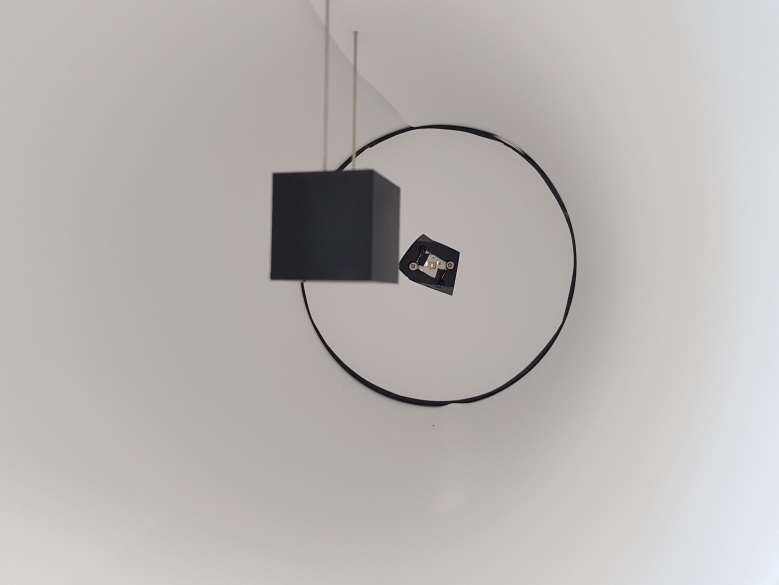
Process challenge device inside of the chamber.

In many cases, objects disinfected in UV-C enclosures are 3D objects. In order to evaluate the effectiveness of the process, it was necessary to design a process challenge device that would simulate a 3D object placed inside of the chamber. For this purpose, a 30 mm × 30 mm × 30 mm cube (Fig. 3) was designed with two 1 mm diameter, 120 mm long stainless-steel rods protruding from its top surface. These rods were used to suspend the sample in the middle of the test chamber, as per Fig. 4.

### 2.3 Exposure Indicators

**Fig. 5:**
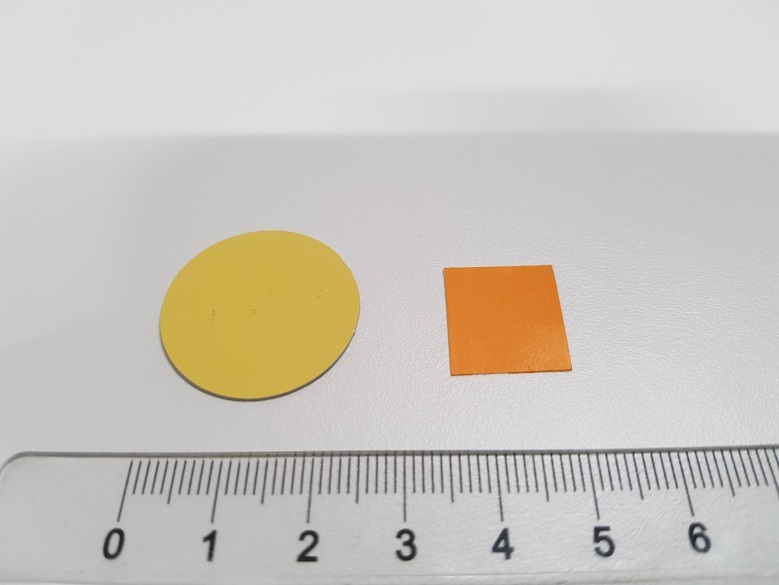
UV-C indicators: Intellego Technologies UVC 100 (left) and Excelsior Scientific CPI-UV1E (right). Ruler is provided for scale purposes in cm.

**Fig. 6:**
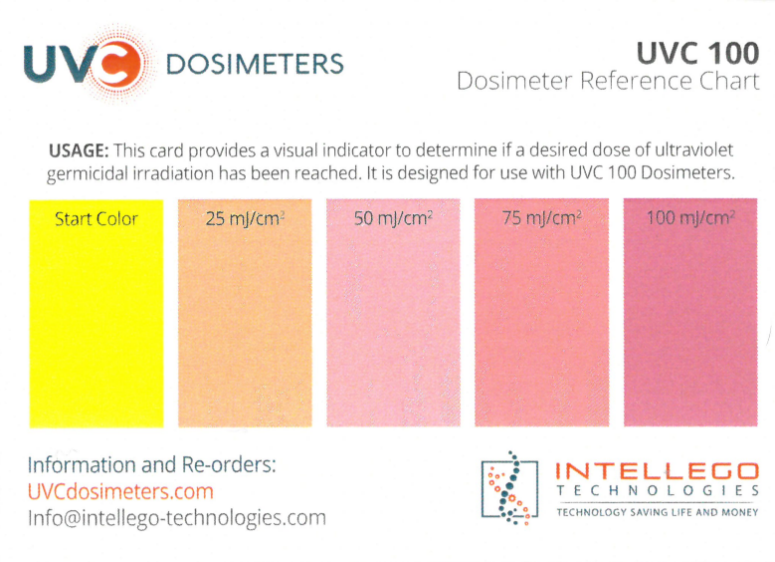
Intellego Technologies UVC 100 reference scale.

Photochromic indicators were proven to be a practical tool for dose validation by Su *et al*. [8]. Figure 5 shows the two types of synthetic, color-changing UV dose indicators that were selected for this study: UVC 100 dosimeter from Intellego Technologies [9] and CPI-UV1E indicator from Excelsior Scientific [10]. Intellego Technologies provided a dosimeter scale (shown in Fig. 6) that links the indicator's color change to specific radiation energy input per square centimeter. This scale was referenced against a 254 nm wavelength UV-C radiation source. At the time of the study, no reference data were available for the CPI-UV1E indicators from Excelsior Scientific. For the purpose of the material comparison, the study focused on the relative difference of the indicator's color change.

Radiant emittance of the LED can be analyzed as a point source, where light disperses from the source in a cone shape. According to manufacturer LiteON, the LED has a maximum solid angle of 120° and a nominal radiant flux of 62 mW at 600 mA and 6.2 V [7].

### 2.4 Experiment Design

**Fig. 7:**
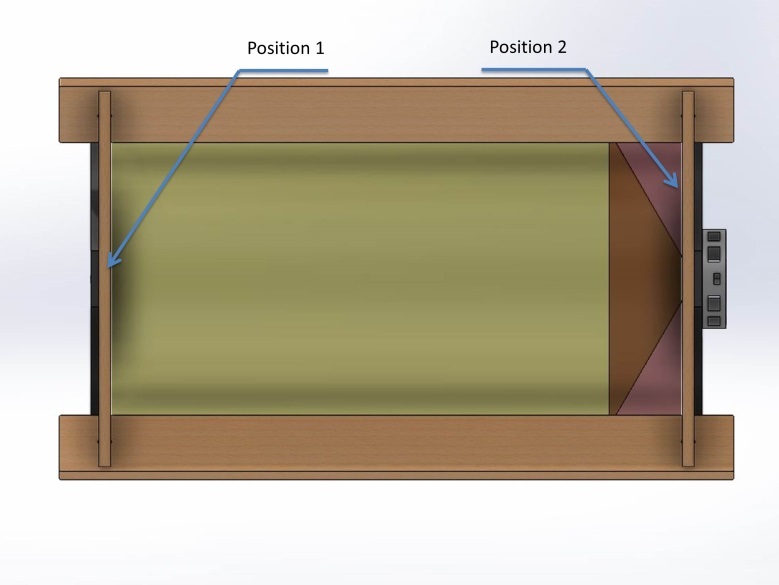
Locations of indicators for the first stage of the study.

The study was separated into two stages. In the first stage, indicators were placed in an empty chamber in two positions. Position 1 was in the middle of the wall directly opposite from the light source at a distance of 300 mm. Position 2 was on the same wall as the light source, which ensured that the indicator was exposed only to indirect light, as per Fig. 7.

Four materials were compared for their reflective properties: white microporous Virtek PMR10 polytetrafluoroethylene (PTFE) sheet supplied by Porex Technologies Ltd. [11], acrylonitrile butadiene styrene (ABS) smooth white plastic supplied by Plastock Ltd. [12], polished gloss self-adhesive aluminum foil supplied by RS (U.K.) [13], and Rosco matte black Cinefoil supplied by Wex Photo Video [14]. The first three materials, especially mirror polished aluminum, are commonly used for the inside lining of enclosures used for UV-C disinfection. Matte black Cinefoil was chosen as a material that was expected to absorb UV-C radiation well and would clearly show the difference between the directly and indirectly exposed indicators.

In the second stage of the study, the cubic process challenge device, suspended in the middle of the chamber as per Fig. 4, was used to compare the two best performing materials from the first stage. In this section of the study, for each cycle, a single Intellego UVC 100 indicator was cut into six pieces and placed on each side of the cube.

## 3 Analysis of the Results

The results of the first stage of the study are presented in Table 1.

**Table 1:** Stage one results of direct and indirect UV-C exposure on Intellego Technologies (left) and Excelsior Scientific (right) indicators for four reflective surfaces. Position 1 represents direct exposure, and position 2 represents indirect eposure to reflected radiation. Results show that PTFE reflected UV-C radiation best.

	Microporous PTFE		Polished Aluminum Foil		ABS		Black Aluminum Foil	
Min	Position 1	Position 2	Position 1	Position 2	Position 1	Position 2	Position 1	Position 2
0	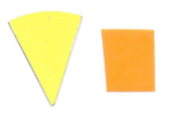	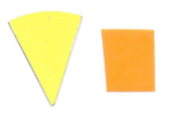	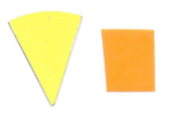	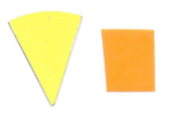	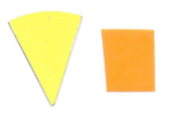	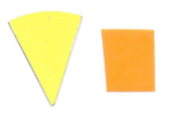	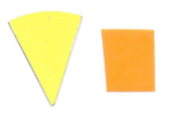	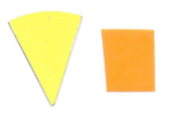
15	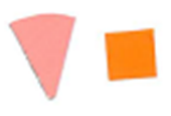	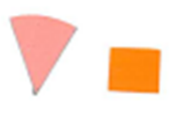	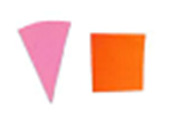	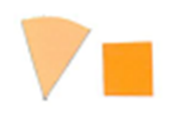	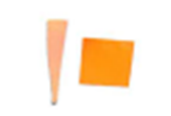	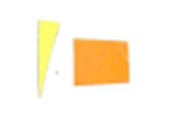	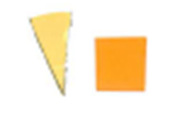	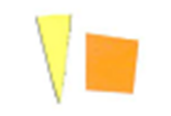
30	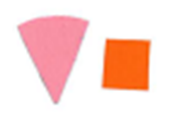	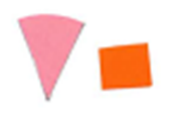	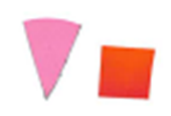	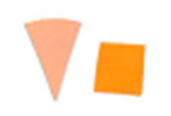	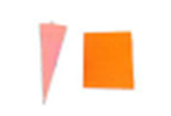	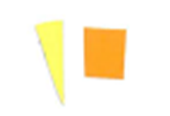	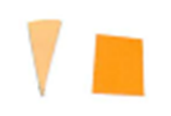	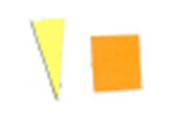
45	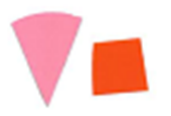	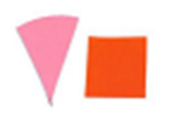	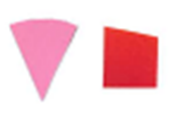	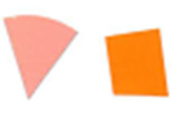	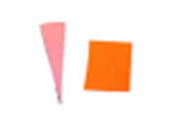	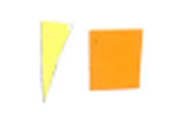	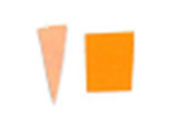	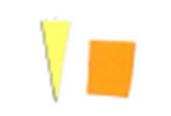
60	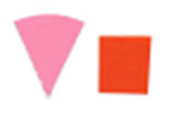	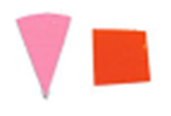	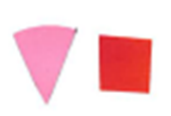	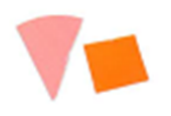	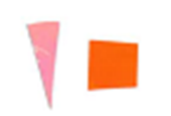	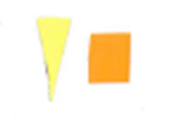	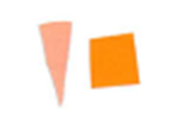	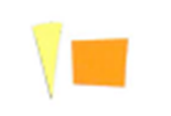

PTFE reflected the UV-C light at 275 nm wavelength most efficiently. Both indicators showed a significant change of color at each time interval. Indicators located in position 2, which were not directly exposed to the light, showed very little difference in color compared to indicators located in position 1. For PTFE, differences in effective dosages between positions were difficult to distinguish with the naked eye.

Polished aluminum foil showed a good amount of color change on indicators in position 1 comparable with PTFE; however, there was a significant difference between positions 1 and 2 for each interval. At 60 min intervals, indicators in position 2 did not achieve the same color change as indicators in position 1 with an exposure of only 15 min. In position 1, under direct exposure, indicators changed color significantly in short periods of time, but it took considerably longer to change color for indicators located in position 2.

For the matte black aluminum foil in position 1, there was a difference in color after each exposure interval; however, even after 60 min, this difference was comparable only to the polished aluminum foil in position 2 after 15 min. Indicators in position 2 did not change color noticeably throughout the entire experiment.

For the ABS, similarly to the matte black aluminum foil, the only difference in color occurred in position 1. Position 2 indicators remained unchanged for all four intervals.

For the second stage of the study with a suspended process challenge device, based on the initial results, PTFE and polished aluminum foil were selected for direct comparison. Table 2 shows the results for the PTFE material.

**Table 2:** Microporous PTFE chamber lining. Intellego UVC 100 indicators located on each wall of the process challenge device.

Time [min]	Front	Back	Top	Bottom	Left	Right
0	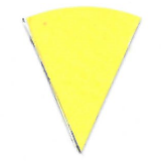	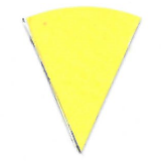	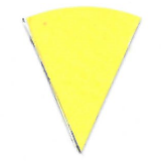	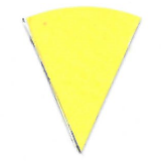	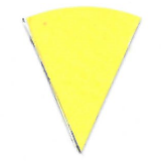	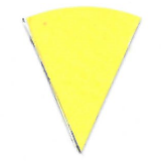
15	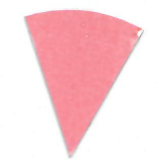	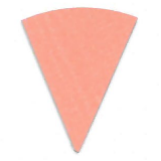	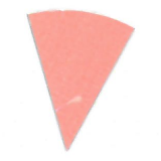	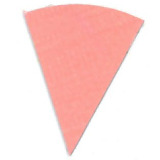	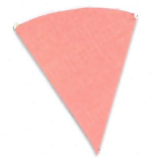	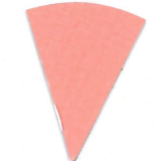
30	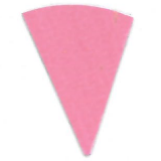	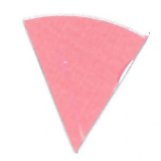	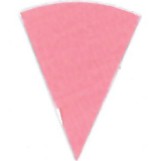	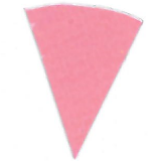	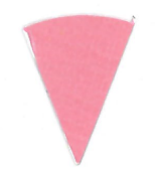	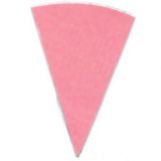

Table 3 shows the results for the polished aluminum foil. Intellego UVC 100 indicators were placed on each side of the cube and exposed to UV-C radiation for different amounts of time, 15 min and 30 min.

**Table 3:** Polished aluminum foil chamber lining. Intellego UVC 100 indicators located on each wall of the process challenge device.

Time [min]	Front	Back	Top	Bottom	Left	Right
0	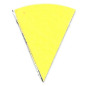	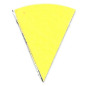	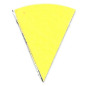	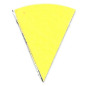	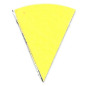	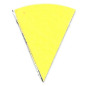
15	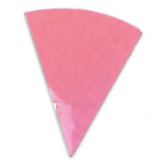	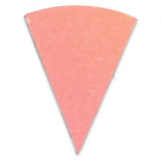	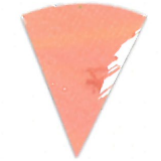	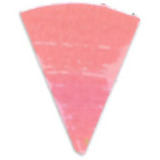	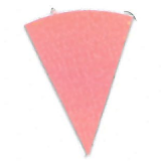	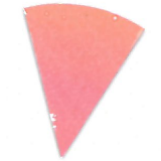
30	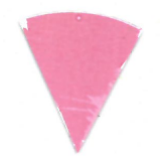	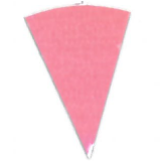	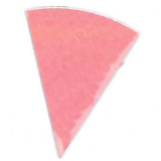	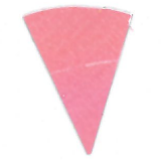	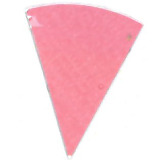	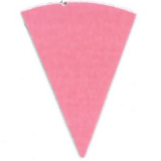

Vertical and horizontal scratches as well as tears on the side of some indicators occurred during their transfer from the process challenge device onto paper that was subsequently scanned to create the images shown here.

**Fig. 8:**
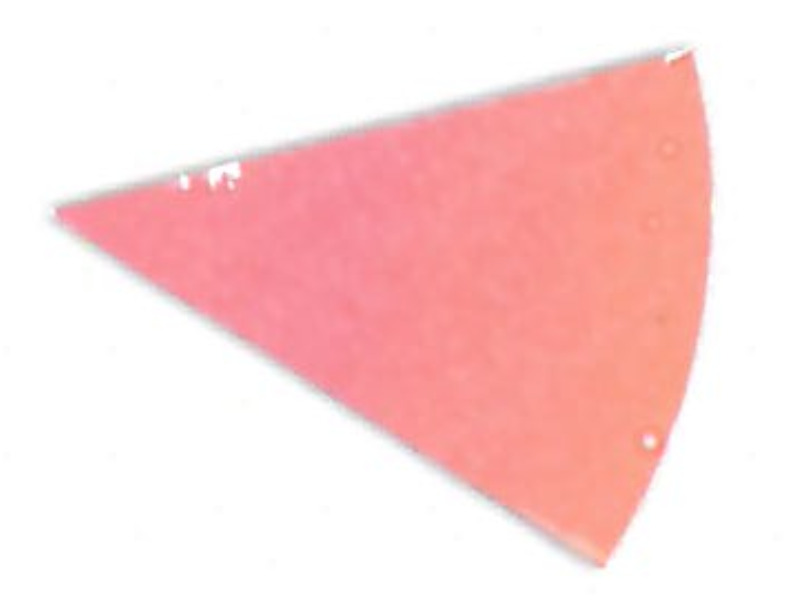
Digital magnification of an indicator from the right side of the process challenge device after 15 min of exposure to UV-C light with polished aluminum foil as the chamber lining material.

**Fig. 9:**
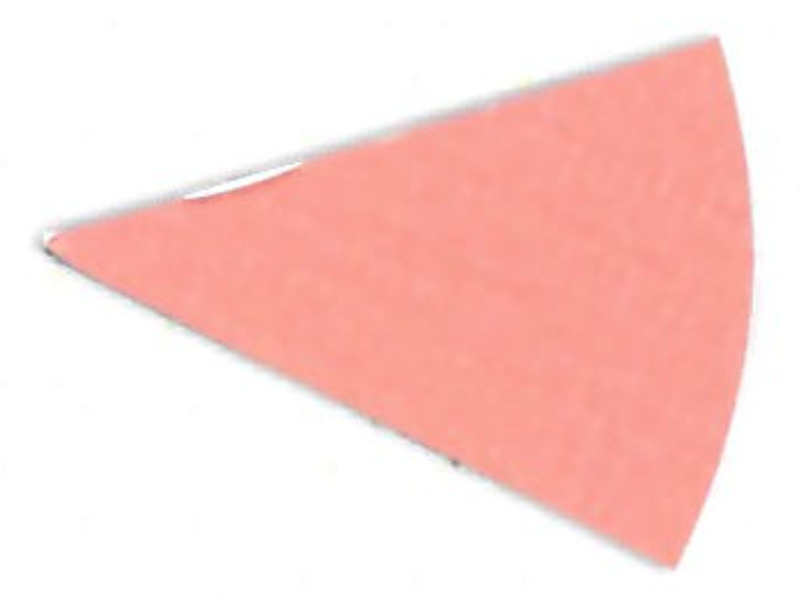
Digital magnification of an indicator from the right side of the process challenge device after 15 min of exposure to UV-C light with PTFE as the chamber lining material.

After transferring indicators onto a white sheet of paper and scanning them, it was possible to magnify the pictures to see in detail the color intensity. No significant difference was observed in color change between indicators for the PTFE test; however, there was a notable effect on some indicators for the aluminum experiments, as seen in Fig. 8 and Fig. 9.

This effect can be described as a gradual change in color intensity. Under magnification, it was possible to see the magnitude of this difference (see Fig. 8). This effect is especially interesting when it is considered that these indicators are only 8 mm to 10 mm in radius. This suggests that polished aluminum foil is prone to creating localized zones of different light intensity, also known as hot and cold spots.

**Fig. 10:**
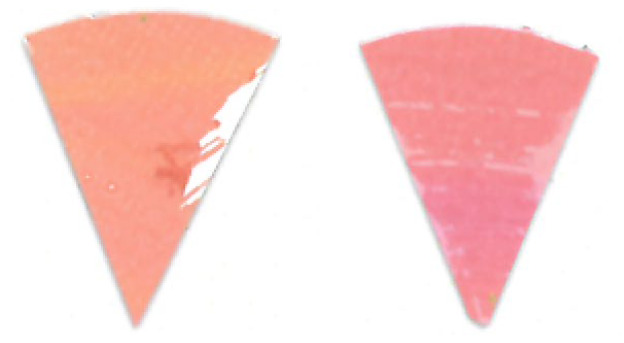
Difference in color between top (left) and bottom (right) positions on a process challenge device in a polished aluminum foil experiment under 15 min exposure to UV-C light.

**Fig. 11:**
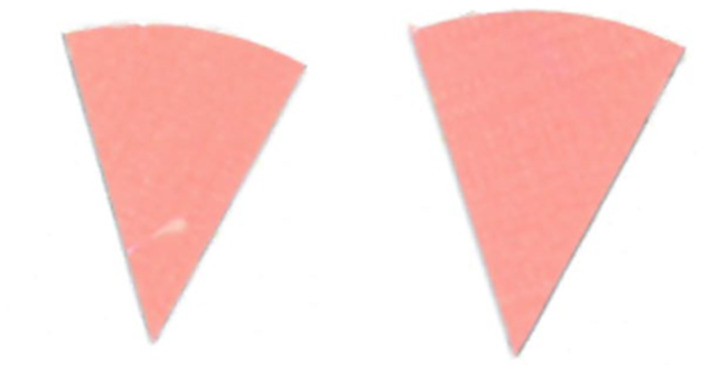
Difference in color between top (left) and bottom (right) positions on a process challenge device in a PTFE experiment under 15 min exposure to UV-C light.

Similarly, as seen in Fig. 10, the difference between indicators placed on the top and bottom surfaces of the process challenge device would suggest there is a significant difference in the uniformity of the inactivation dose reaching these two surfaces when polished aluminum foil is used as the chamber lining material. It can be hypothesized that the two mounting stainless-steel rods may deflect the light on the top surface and create additional shadows, but the same effect was not observed when the chamber was lined with PTFE. As seen in Fig. 11, a similar effect is not visible by the naked eye when PTFE was used as the lining material.

## 4 Conclusions

Differences between the ability of the chosen materials to reflect UV-C radiation were significant. Of the four materials tested in the first stage of the study, the microporous PTFE sheet had the highest reflectivity of UV-C radiation, as demonstrated by no significant difference between indicators in positions 1 and 2 across all chosen time intervals. This suggests that UV-C radiation was being reflected and scattered evenly inside of the chamber. Polished aluminum foil also reflected UV-C radiation but not as well as the PTFE sheet, and the difference between directly and indirectly exposed indicators was significant. The ABS sheet, despite reflecting the visual spectrum of light well, was more similar to matte black aluminum foil when subjected to UV-C. Both materials, despite their obvious physical difference in color, did not reflect UV-C radiation well. This was confirmed by no significant change in the color of indicators in position 2, even under 60 min exposure time.

From the perspective of materials used to line the internal walls of disinfecting chambers, the reflectivity of the material is critical to ensure that complex-shaped objects get uniformly exposed to the UV-C radiation. From the materials tested in this work, only the PTFE sheet and polished aluminum foil are suitable materials for this application, with the caveat that in some areas covered only by the reflected light, polished aluminum foil would require roughly four times the amount of time to achieve the same effect as the PTFE sheet with the same applied light power.

The difference in the exposure time required to significantly change the color of indicators in positions 1 and 2 among different materials suggests that, in the case of PTFE, the material is not only highly reflective but also scatters light effectively, which ensures effective diffusion of the disinfecting radiation dose.

It was noted that with the polished aluminum foil coverage, the UV-C dose achieved inside the chamber and on the challenge device was not uniform. Even on a small surface area, such as the indicators used in the study, certain locations presented a gradient of color that was visible even to the naked eye. Such a gradient would be expected to be even bigger on complex parts, with shadowing exacerbating the effect. In the same experiment using polished aluminum foil, under 15 min of exposure, the differences in exposure of indicators on the top and bottom as well as left and right sides of the process challenge device were clearly visible. The PTFE material used in the experiment did not show the same behavior.

The premise of this study was to use indicators only and rely on visual inspection, since such a method is much simpler and might be used by manufacturers for simple evaluation and comparison of different designs. Potential future studies could repeat the experiment and measure the color change accurately, such as with the use of a photometric analyzer, but the aim of this study was to compare the chamber lining materials and differences shown in the papers evaluated. These changes were significant enough to be observed by simple visual inspection.
